# Computerized Simulation Education on Physiotherapy Students' Skills and Knowledge: A Systematic Review

**DOI:** 10.1155/2022/4552974

**Published:** 2022-10-26

**Authors:** Sorayya Rezayi, Leila Shahmoradi, Nastaran Ghotbi, Haniyeh Choobsaz, Mohaddeseh Hafez Yousefi, Shahab Pourazadi, Zakiyeh Raisi Ardali

**Affiliations:** ^1^Health Information Management and Medical Informatics Department, School of Allied Medical Sciences, Tehran University of Medical Sciences, Tehran, Iran; ^2^Department of Physiotherapy, School of Rehabilitation, Tehran University of Medical Sciences, Tehran, Iran; ^3^Advanced Intelligent Systems Robotics Company, Vancouver, British Columbia, Canada

## Abstract

**Introduction:**

Applying computerized simulation education tool for learning in medical domains is widely used in many countries. This review is aimed at systematically investigating the computerized simulation tools developed to educate physiotherapy students and determine the effectiveness of these interventions.

**Methods:**

A comprehensive search was conducted in Medline (through PubMed) and Scopus databases from inception to Sept. 10, 2022. The studies that examined the effectiveness of computerized simulation-based interventions were included.

**Results:**

Sixteen studies were included in this systematic review. All included examinations were ranked “good” or “low risk of bias” based on the criteria utilized in the Joanna Briggs Institute (JBI) scale and the Effective Public Health Practice Project (EPHPP) tool. Most of the articles (43%) were conducted in the USA and 25% in Australia. In 43% of the total studies, the study population was only physiotherapy students, and in 12.5% of them, the scope of education was related to practical skills training. Three of the 16 reviewed articles presented positive qualitative results; thirteen quantitative investigations also declared statistically positive effects. Positive effects have been seen in areas such as improving professional and behavioral abilities, improving knowledge and self-confidence, and reducing stress. The sample size of the studies ranged from eight to 162 participants. The limited sample sizes in groups, lack of interaction, and short follow-up duration were the most consistent limitations evident within the included studies.

**Conclusion:**

Computerized simulation education approaches can help to improve physiotherapy students' skills and knowledge. They also have great potential to reduce learning costs and increase the quality of education.

## 1. Introduction

Clinical education is a critical component of physiotherapy student education that, using a centralized and structured process, can expose students to a variety of training opportunities to develop clinical learning [1] and is defined as providing guidance and feedback on the trainee's personal, professional, and educational progress in delivering appropriate patient care [2]. Students experience their most effective clinical-operational learning in places where there is a relaxed atmosphere between students and the clinical instructor to ask questions and receive feedback from their professors [3, 4]. Furthermore, a fundamental challenge in the clinical education of physiotherapy students, given the importance of gaining clinical experience, is the demand for trainer supervision on the student's performance during the interventions of patients [5]. Due to students' limited years of study, clinical education cannot be provided in a safe environment for a wide range of diseases [6]. Historically, practical skills in physiotherapy curricula have been instructed via live demonstration, followed by activity and feedback, in a manner and time specified by constraints [7]. This leaves students to revise the skill outside class time based on memory or handwritten, potentially inaccurate notes. As such, practical skills development may not have been optimal [3, 8]. A structured clinical education program with teaching and learning activities can facilitate the quality of clinical education. The students' learning experience is enhanced by a learning environment enriched with visual and cognitive modeling [9].

Therefore, the existence of tools that can, in addition to active student learning, allow repetition of exercises in a fun environment and create ongoing education will improve the quality of medical services furnished by students to patients [10]. Computer simulations can improve physiotherapy students' skills in patient assessment, treatment, and clinical decision-making [9, 11]. One of the new techniques is simulation-based education, which is widely operated in various areas of the healthcare system [12]. In recent years, simulation has become a standard practice in teaching technical skills in the physiotherapy field [13]. Scientifically speaking, these technologies, which operate on a connection basis at any time and place, have significantly influenced healthcare measures. Simulation-based teaching and learning also breaks down time and space constraints and enables one to use educational programs in any setting [14]. The benefits of using clinical education simulation include improving technical and communication knowledge and skills, increasing student satisfaction, and improving clinical decision-making that allows the student to gain the right clinical experience in a safe and controlled environment [15]. Remarkably, simulation never completely replaces real learning experiences in the clinical setting [16].

Multifarious simulation techniques reported in reviewed physiotherapy research include simulated patients (classmates, actors, or volunteers trained to demonstrate the role of a patient called a standard patient), pictures, video, computer simulators, web-based learning, designed software for specific occasions, virtual reality simulators, and mannequins [17, 18]. Computer technology allows students to explicitly develop metacognition, reflecting on their own learning, improving their motivation and interest in the classroom, and presenting themselves as an effective predictive tool [19]. It is noteworthy that computer simulation tools such as virtual reality, augmented reality, and web-based simulations offer a wide variety of opportunities for modeling concepts and processes [20]. These new simulation technologies bridge the gap between prior and unique knowledge of physiotherapy students, learn new clinical operations, and help them develop their scientific understanding using knowledge in a quasirealistic environment [21]. Therefore, it would be safe to say that these simulated tools are considered new approaches that strengthen students' skills in specific areas such as attention and perceptual abilities; immersion in these environments improves visual and auditory feedback [22, 23]. These technologies have reasonable potential to create scenarios for physiotherapy students in the field of interactive learning with patients and their treatment, which provide organizing clinical education [24]. In recent years, several systematic reviews have been conducted to investigate the effects of using simulated environments for physiotherapists and students. The authors related to these articles have claimed that by leaving aside some problems of designing and developing some simulation tools for the training of physiotherapists, significant effects have been seen in the related skills [25–27]. Roberts and Cooper [28] concluded that physiotherapy students did not improve their communication skills, and no significant difference was observed in practical skills. In terms of clinical performance, the Physical Therapy Practice Assessment Tool (APP) did not significantly increase mean scores. Likewise, it was observed that computerized mannequins improve students' preparedness for clinical practice but do not improve students' clinical performance or skills [29]. Due to some inconsistencies in the results, it was decided to do a more recent review of the studies. The goal was to review quantitative and qualitative articles comprehensively so that we could answer research questions.

### 1.1. Objective

Technology-based simulated education settings for learning physiotherapy students are widely used in many countries, and the benefits and effects of this technology-based education have been noted in most published articles; for example, clinical and operational knowledge and students' self-confidence have been declared improved [8, 30, 31]. In this qualitative literature review, findings about computerized simulation education on physiotherapy students' skills and knowledge were summarized and synthesized. The main questions and ambiguities of this review are as follows:

- Generally, how many articles have been published in the field of investigating the effect of computerized simulation education on physiotherapy students' skills and knowledge (what is the publication trend)?
- What are the main features of the studies, i.e., study aim, training tool, the scope of education, study place, study design, participant's description (sex and age (year)), key results, critical effects, effectiveness, main message, study limitations, and barriers for the use of technologies?
- How successful has computerized simulation education been reported on physiotherapy students' skills and knowledge?
- How is the studies' risk of bias? With what tool/tools is this index measured?

## 2. Materials and Methods

This systematic review (SR) was conducted based on the JBI framework. The main steps are the followings: (1) planning; (2) identification; (3) screening; (4) eligibility/assessment, and (5) presentation (synopsis of findings, discussion, and presentation of the results) [32]. Also, a qualitative analysis process was used to summarize the reviewed studies and generate new remarkable insights. Reporting of this SR is based on the Preferred Reporting Items for Systematic Review and Meta-analysis (PRISMA) statement [33]. The filled PRISMA checklist is given as the supplementary material (Appendix Table S1).

### 2.1. Eligibility Criteria

The PICO model (patient/population, intervention, comparison, and outcomes) was used to explore main queries and facilitate literature review. Various inclusion and exclusion criteria were adjusted in this review which are presented below.

#### 2.1.1. Inclusion Criteria

**Figure 1:**
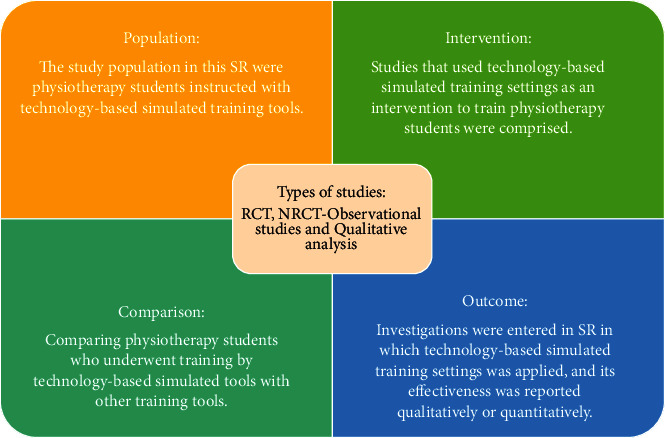
Inclusion criteria in this SR.

Figure 1 depicts the inclusion criteria that were admitted in this review.

#### 2.1.2. Exclusion Criteria

The exclusion criteria were as follows:

- Studies related to education without simulation
- Studies related to the field of treatment
- Simulation studies in areas other than physiotherapy
- Noninterventional studies
- Studies with standard patient simulation, role-playing, robotics, and mannequin
- Conference papers
- Studies in which the target group was physiotherapy graduates were excluded
- Non-English papers

### 2.2. Information Sources and Search Strategy

Electronic search strategies were performed via Medline (through PubMed) and Scopus to identify papers from inception to Sept. 10, 2022. The search strategy used in this SR included a combination of keywords and Medical Subject Headings (Mesh) terms related to “Physiotherapy,” “Education,” “Virtual Reality,” “Augmented Reality,” “Computer Simulation,” and “Simulation Training.” The complete list of keywords and terms used in the search strategy for Scopus and PubMed databases is given in the supplementary material (Appendix Table S2).

### 2.3. Study Selection

**Figure 2:**
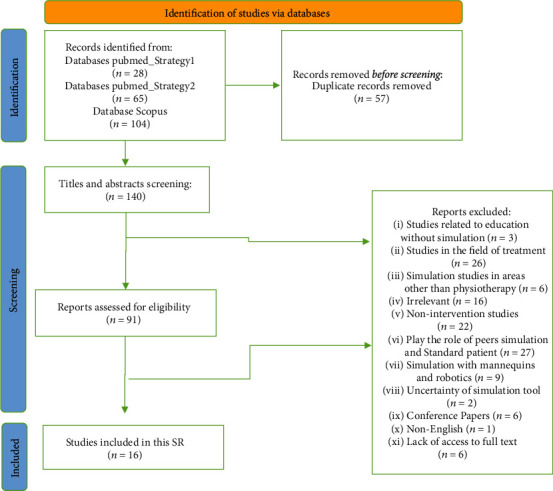
The flow diagram based on PRISMA for determining, selecting, and mining of included papers.

Two stages were conducted in the selection process. Five reviewers (SR/ZR/HC/MY/SP) independently screened the abstracts and titles of the retrieved papers in the first stage; in this phase, papers that did not meet the eligibility criteria were extracted. The citations' full text was screened and inspected in the second stage, and three reviewers confirmed their relevance. The supervisors (LS and NG) were consulted in a disagreement not solved by a consensus discussion. The screening process is depicted by the 2020 PRISMA checklist in Figure 2.

### 2.4. Data Collection Process and Data Items

Five reviewers gathered the required information from the included papers. Then, two other reviewers ascertained the accuracy of the information accumulated. Any dissensions were examined and resolved with reviewers (LS/NG). A form in Excel was prepared to extract data from included articles. The following data were extracted for each citation: publication details, study aim, training tool, the scope of education, study place, study design, participant's description (sex and age (year)), key results, key effects, effectiveness, main message, study limitations, and barriers for the use of technologies. The effect of applied simulation-based learning was summarized into three classes: (1) statistically significant effect, (2) effective without statistical argument, and (3) without any effectiveness (not statistically significant).

### 2.5. Study Risk of Bias Assessment

For qualitative studies, the JBI critical appraisal checklist was chosen to evaluate articles' quality and risk of bias. Furthermore, the EPHPP tool for qualitative ones was also used to evaluate the included papers. These tools were applied to evaluate all types of studies' methodological conduct or reporting. The JBI tool for qualitative studies has ten questions; these can be answered with four choices: (1) yes, (2) no, (3) unclear, and (4) not applicable. Each “yes” answer reaches one score, and if 70% of the questions responded “yes” in research, the risk of bias was considered “low,” and if 50%-69% of them were answered yes, the risk of bias was supposed “moderate,” and ultimately below 50% considered “high risk” 24. However, the EPHPP checklist was chosen because it assesses various quantitative studies' quality. The risk of bias was determined for six elements in each study: (1) selection bias, (2) study design, (3) confounders, (4) blinding, (5) data collection method, and (6) withdrawals and dropouts [34, 35]. These six components were rated on a strong, moderate, and weak three-point scale; overall quality is ranked as a score of weak (two or more poor ranking of individual scale), moderate (one weak individual scale rating), and strong (no weak scale rating). Each study was investigated by three authors (SR/HC/MY) for bias and quality assessment by tools as mentioned earlier—JBI and EPHPP—and any disagreements were resolved by discussion with LS and NG.

### 2.6. Synthesis of Results

In this SR, as the procedures and methodology of reporting consequences in selected articles were heterogeneous, meta-analysis was not accomplished.

## 3. Results

### 3.1. Study Selection

A total of 197 articles were found in the initial search; after removing duplicates, 140 papers were left. Authors examined the title, abstracts of selected articles, and keywords, so 91 articles were identified for further review. After viewing the full text of these articles and focusing on simulation tools for physiotherapy in the field of clinical education and applying inclusion criteria, finally, 16 articles were included in this SR. Based on the predefined classification elements, a summary of the key classifications is described in Table 1.

### 3.2. Quality of Studies

**Figure 3:**
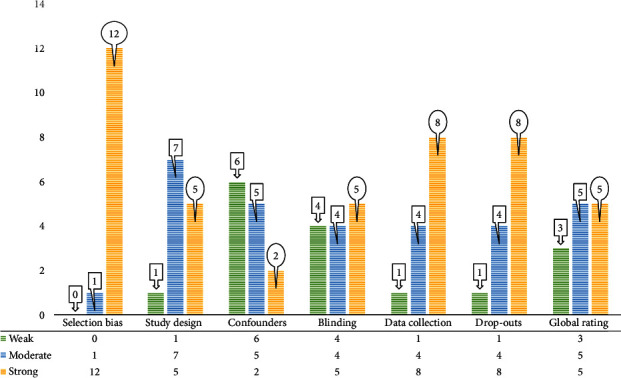
Quality assessment of the twelve quantitative included studies.

In this SR, quantitative and qualitative studies with different study designs are included, and based on it, they were evaluated with various appropriate scales. The EPHPP tool was used to assess the quantitative studies, while the JBI checklist was used for qualitative studies. Of the three qualitative studies that were evaluated with the tool JBI, all three were judged as with a low risk of bias (Table 2). Thirteen studies were evaluated by the EPHPP checklist. Also, the results of quality assessment for qualitative studies are shown in Figure 3. Based on the sum of scores, most studies were strong in terms of selection bias (92%), data collection (61%), drop-out (61%), and moderate in terms of study design (53%). Concerning the global rating, 38% of the thirteen quantitative included studies were strong, 38% moderate, and 23% weak. It can be concluded that all included examinations were ranked “good” or “low risk of bias” based on the criteria utilized in the JBI scale and EPHPP tool.

### 3.3. General Characteristics of the Included Studies

**Figure 4:**
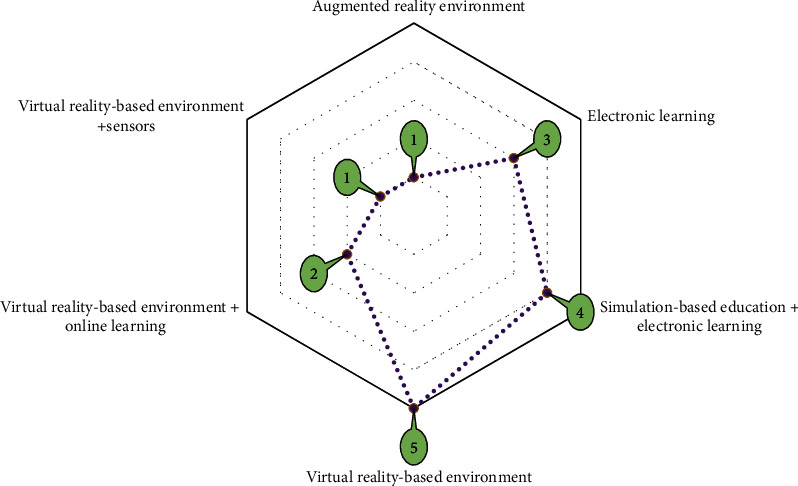
Distribution of studies based on training tool frameworks.

Of 16 included papers, the oldest and newest papers were published in 2008 and 2022, respectively. Most of the papers (43%) were conducted in the USA, 25% in Australia, and 18% in Sweden.  Table 3 shows the frequency distribution of the study population based on the scope of education. In 43% of the total studies, the study population was physiotherapy students, and in 12.5% of them, the scope of education was related to practical skills training. Also, in 12.5% of the total studies, the study population was physiotherapy and occupational therapy students, and in all of them, the scope of education was in terms of interprofessional skills. In five studies (31%) of the total citations, the scope of education and learning was related to improving interprofessional skills, and also, in two of them (12%), the scope of education was enhancing practical skills. The sample size of the studies ranged from 8 to 162 participants (IQR1: 29, median: 56, and IQR3: 69). Training tool frameworks in included papers are classified into six main categories.  Figure 4 presents the distribution of studies by training tools frameworks.

### 3.4. Effects of Computerized Simulation Education on Physiotherapy Students

Table 4 presents the effects of applying computerized simulation education. The effects of computerized simulation education on physiotherapy students were classified into two groups: (1) professional skills, behaviors, and knowledge and (2) physiotherapist-reported effects. Most papers had reported more than one effect. Three of the 16 reviewed articles presented qualitative results that reported positive effects without statistical analysis or arguments. Thirteen of the quantitative investigations also declared statistically positive effects. It is noteworthy that we did not have a study that did not report a relatively positive effect.

### 3.5. Reported Limitations and Barriers to Applying Technologies

Most examinations reported limitations related to conducting studies. In fact, the limitations of conducting studies and implementation barriers attributed to the limited use and effectiveness of computer simulation education from the authors' point of view are given in detail in Table 5.

## 4. Discussion

### 4.1. Findings

This study is aimed at evaluating the effectiveness of computer simulation education on the skills and knowledge of physiotherapy students. To this end, sixteen studies on the effect of computer simulation were systematically reviewed. Thirteen studies were evaluated by the EPHPP checklist. The quality evaluation results for quantitative studies have been shown to be 38% strong, 38% moderate, and 23% weak. Also, three qualitative studies evaluated by the JBI were judged as with a low risk of bias.

In most studies, physiotherapy students reported the positive effect of computerized simulation methods on improving basic knowledge, clinical reasoning, and practical and interprofessional communication skills. Students' learning levels improved after participating in computerized simulation courses; it was reported that they reached more motivation and self-efficacy. Also, the dependency on educators with these programs was diminished.

Many studies in the educational scope were related to improving cardiopulmonary auscultation and interprofessional skills. Also, neurologic physiotherapy, cultural empathy, practical skills, and pediatric clinical training were investigated. Numerous studies have addressed the importance of interprofessional education. Paying attention to this issue as one of the essential educational areas can lead students to the goal of two-way education in different domains of study and helps to understand the content more deeply; on the other hand, accomplishing interprofessional education in congested university environments can be challenging [36]. A technology-based computer platform enables interprofessional interaction in a customized space to display an educational-clinical environment and has the potential to bring students together in more flexible cyberspace rather than enclosing an academic classroom [37].

According to the results of this SR, most studies (43%) were conducted in the United States and (25%) in Australia. It seems that developed countries face time constraints in face-to-face clinical education courses. In medical systems, reducing patients' length of hospital stay to reduce the risk of nosocomial infections is also considered [11]. These issues can reduce the emphasis on face-to-face education for medical students. Therefore, computer simulation education in many developed countries has replaced traditional methods to increase clinical learning opportunities.

Within studies included in this SR, the authors have reported some limitations: the cost of technology, the time-consuming work with technology, and the need to allocate a suitable physical location were among the most critical mentioned issues. It should be noted that depending on the practical details of the systems and the objectives of the study, the amount of cost and required time and implementation methods have been reported differently, and on the other hand, the inflexibility of the systems is a significant limitation. Other limitations include the demand for long-term evaluations (evaluating the effects of long-term education), problems with Internet access for online education, and the time-consuming technology training, as well as the need for sufficient knowledge to become familiar with technology; the presence of the operator has also been expressed in some investigations.

In the present SR, the framework of educational tools in the included studies comprises virtual reality-based environments (31%) and simulation-based e-learning (25%). Also, e-learning, virtual reality-based environment, online learning, and virtual reality-based environment plus sensor have been employed.

### 4.2. Interpretation of Results

In recent years, several systematic reviews have been conducted to evaluate the effects of simulation tools on physiotherapy students' practices. In some reviews, authors have declared effectiveness on several or all scales [25, 38], but in others, significant effects were not observed [27–29]. However, this SR's results demonstrated that in most studies, physiotherapy students reported a positive effect of computer simulation methods on improving clinical reasoning, basic knowledge, and practical skills.

The last review study was designed in 2020 to “ examine the role of the simulated patient in physiotherapy education,” and its results indicated that the simulated patient is a valuable learning strategy for providing educational activities in medical education and physiotherapy curricula [39]. The simulated patient also facilitates student performance feedback to interact with patients and real-world environments. In the beforementioned review, the included studies acknowledged that the simulated patients could be used for various purposes, including education, improving self-perception skills, clinical practice, and expanding the attitudes of physiotherapy students. They also argued that simulated patients as an educational technique could improve physiotherapy students' clinical reasoning, communication skills, and motivations.

As far as we know, this is the first SR to evaluate the effectiveness of with emphasis on computer simulation education on the skills and knowledge of physiotherapy. Our results, in line with previous studies, emphasized that evaluation methods that consider communication education efficacy and effectiveness need to be improved to increase education quality [25]. Our study showed that computer simulation, especially if appropriately utilized for instructional delivery, will enhance learning and improve physiotherapy student performance as it may allow students to observe and visualize the step-by-step actions and reactions that take place in any treatment process. It seems that computer simulation can be a suitable method to replace the traditional simulation method. One possibility is that today, computer simulation education, especially for young student generations who are more prominent in adapting to new technologies and increasingly familiarized with computers, encourages them to further development and improvements in this field to introduce education with more fun.

In our study, we emphasized physiotherapy students because experienced physiotherapist therapists are familiar with the use of highly complex virtual reality systems as an intervention to use with clients' reports; it may be necessary that it is used and updated in curricula for teaching discipline-specific and interprofessional skills [40].

Also, in 2015, Mori et al. examined the impact of simulation-based learning activities in physiotherapy curricula using a full range of simulation techniques such as virtual reality, role-playing, written scenarios, and mannequins. Simulation learning experiences in physiotherapy students showed that simulation methods effectively facilitate the development of assessment skills, attitudes, and clinical reasoning of assessment students and can be included in physiotherapy curricula [25].

### 4.3. Strengths and Limitations

This study is aimed at evaluating the effectiveness of computer simulation in the education of physiotherapy students; due to the vital education category, only studies that evaluated students were fitted in the SR, and examinations that incorporated experienced physiotherapists were excluded. This diversity and heterogeneity of studies limited the number of input articles. Overall, the central gap was the heterogeneity of the included studies. In the future, a review can be conducted that will include the target group of physiotherapists and physiotherapy students. Another limitation was the lack of generalization of the effects on appropriate physiotherapy training. In the current SR, studies that evaluated physiotherapy students at all levels of education, including BSc (Bachelor of Sciences), MSc (Master of Sciences), and DPT (Doctor of Physiotherapy), were included. However, some papers did not mention the student's degree. It seems that due to the different levels of knowledge of individuals and their attitudes towards education, it is better to pay more attention to the level of education of physiotherapy students.

On the other hand, due to the prevalence of COVID-19 as a pandemic, governments have shut down several activities across the country, including face-to-face training. This has led to the desire of universities to teach online as a learning platform, and learning computer-based simulation has become a significant challenge [41]. Given that virtual learning is widely used in physiotherapy as one of the essential disciplines of medical sciences, it seems that moving towards virtual simulation-based education, especially computer simulation education, is necessary for the future.

### 4.4. Implications for Practice

Computerized-based learning tools have replaced traditional teaching approaches and learning methods recently. Due to the growing global need to use computer simulations, this issue extends significantly in modern countries. Therefore, it is suggested that developing countries also provide a suitable platform for these studies. However, the cost of technology, time, place, and implementation methods vary greatly depending on the details of the application, but most of them are expensive and require enough space. Therefore, it is recommended that governments plan for this type of training and related expenditures.

## 5. Conclusion

This SR highlights the effects of using computer simulation education on the skills and knowledge of physiotherapy students. The survey explained that computer-based simulation solutions had significant potential to improve physiotherapy students' skills and knowledge. e-Learning, virtual reality-based environment, online learning, and virtual reality-based environment plus sensor have been employed in included papers. The principal effectiveness is improving professional skills, behaviors, knowledge, and physiotherapist-reported outcomes like learning practice change, increasing confidence, and motivation reported in citations.

## Figures and Tables

**Table 1:** Summary of descriptive characteristics of the included papers in this SR.

Contribution	Study aim	Training tool	Scope of education	Study population	Study design	Participants description (sex and age (year))	Key results	Main message	Key effects	Platform picture
Björklund and Silén, Sweden. [42].	To explore occupational therapist and physiotherapist students learning of skills in interprofessional communication by studying the student's communication while working together with a virtual patient	Virtual patient	Interprofessional skills	Occupational therapy and physiotherapy students	Qualitative design	PTs *n* = 8 Sex = (*F* = 7, *M* = 1) Age = 21-24 y	Students who had access to online e-learning resources for physiotherapy skills in addition to regular education improved their practical skills, and students found this resource very useful for learning	Working in cooperation in a virtual setting makes facilitate interprofessional communication in students and helps to learn their own profession. An environment was created in which students were able to talk about their careers and gradually reach interprofessional meanings. Communicating and making shared decisions about a patient can facilitate learning how to communicate interprofessional and improve students' understanding of their own profession	♦ Communication interprofessional ♦ Practical skills	Without picture
Preston et al., Australia. [8].	Examine whether the eSkills physiotherapy online training resource improves the performance of practical skills in physiotherapy students in addition to regular training	E-learning	Practical skills training	Physiotherapy students	Nonrandomized controlled trial	PTs Experimental *n* = 35 Sex = (*F* = 22, *M* = 13) Age = 25 (2.3) y Control *n* = 34 Sex = (*F* = 16, *M* = 8) Age = 26 (2.4) y	The experimental group rated the physiotherapy eSkills training online resource for as follows: (1) Improving their practical skills as 8.4 out of 10 (95% CI 8.0 to 8.7) (2) Helping in examination preparation as 8.7 out of 10 (95% CI 8.3 to 9.1) (3) Helping on neurological clinical practicum as 7.6 out of 10 (95% CI 7.0 to 8.2) (4) Usefulness as a new graduate as 6.6 out of 10 (95% CI 5.8 to 7.4)	Students who had access to online e-learning resources for physiotherapy skills in addition to regular education improved their practical skills, and students found this resource very useful for learning	♦ Empathy skill ♦ Physical awareness	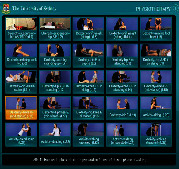
Sabus et al., USA. [36].	To understand the utility of a web-based virtual environment for an interprofessional instructional activity	Web-based 3D virtual reality	Interprofessional skills	Occupational therapy and physiotherapy students	Case study	PTs (*n* = 34), OTs (*n* = 35) Age = 22-25 y	Students consider cyberspace to support learning and support interprofessional collaboration	Students consider cyberspace to support learning and support interprofessional collaboration	♦ Communication interprofessional ♦ Learning ♦ Personal behavior and attitudes	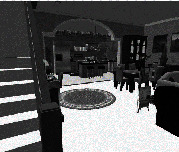
Shahmoradi et al., Iran. [43].	The purposes of this research were to design a VR game and to apply it to teach physiotherapy in neurological diseases	Virtual reality-based game with the Kinect sensor (Xbox 360)	Nervous physiotherapy training	Physiotherapy students	Clinical trial	PTs (*n* = 31) Sex = (*M* = 10, *F* = 21)	Evaluate the facilitating level of the game in education (3 Q): mean (SD) and median of 8.04 (2.18) and 8.66 satisfaction in using the game in education (4 Q) mean (SD) and median 0.27 (2.07) and 910 (*Q*) mean (SD) and median of the 1.79 and 1, respectively	The mean score of students “perception of learning was the level of virtual reality facilitator and student satisfaction was high and the analysis of students” answers to the final questions highlights the therapeutic aspect of the game in comparison with the educational aspect	♦ Learning practice change ♦ Major-related knowledge ♦ Interpersonal skills	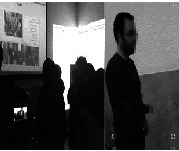
Sword et al., USA. [44].	To describe a novel, convenient, and cost-effective method for evaluating cardiopulmonary auscultation competency in student physical therapists	Mannequin and computer	Cardiopulmonary auscultation skills	Physiotherapy students	Cross-sectional	PTs (*n* = 62) third year	62 students taking and completing the OSCE, 95% (59/62) correctly located the heart auscultation sites and identified the prerecorded heart sound on their first attempt. Ninety-five percent (59/62) also correctly located the pulmonary auscultation sites and correctly identified breath sounds on their first attempt as well. The post-OSCE survey revealed that 75% of students agreed or strongly agreed that this manner of evaluation was an effective way to assess their auscultation skills, increased their confidence with auscultation, and should be included in future OSCEs	The simulation method described in this article is a cost-effective and sustainable tool for assessing hearing competence not only in physiotherapy students but also in other health professions	♦ Interpersonal skills ♦ Practical skills	Without picture
Tran et al., Sweden. [20].	To develop and evaluate a virtual patient model for primary healthcare and to examine students' perceptions with this interprofessional virtual patient model	Virtual patient (film)	Interprofessional skills	Nursing, physiotherapy, medicine, and occupational therapy students	Qualitative design	*n* = 39 Sex = (F = 26, M = 13) Age = 20-46 y (median = 28) Medical (*n* = 12) Sex = (*M* = 7, F = 5) Nursing (*n* = 16) Sex = (M = 3, F = 13) PT (*n* = 4) Sex = (*M* = 2, *F* = 2) OT (*n* = 7) Sex = (*M* = 1, *F* = 6)	VP model facilitated interactions and discussions between students, and the students valued the comprehensive information about different professions' roles via the video clips and written texts. VP model might be a suitable tool for preparing students for future teamwork in clinical practice	The virtual patient model facilitated and used the learning process. It also helped students understand the roles and competencies of professionals and other professions	♦ Communication interprofessional ♦ Learning	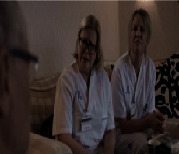
Ward et al., Australia. [45].	To investigate changes in the intrapersonal and interpersonal cultural empathy of final year physiotherapy students in response to a virtual cultural simulation experience and guided reflection and to explore the satisfaction of students regarding this learning experience	Self-directed online virtual simulation (film): eSimulations' (online modules)	Cultural empathy training	Physiotherapy students	Before and after trial	PTS *n* = 162 Age = 18 − 24 y Sex = (*F* = 74%, *M* = 26%) CCQpre: *n* = 84 Sex: (*F* = 55%, *M* = 45%) Age: 23.3 ± 2.8 y CCQpost: *n* = 63 Sex = (*F* = 57%, *M* = 43%) Age = 24.3 ± 4.9	Students reported high satisfaction with the learning experience, with a mean total score of 40/56 (71%)	A virtual cultural simulation experience and guided reflection led to significant increases in students' intrapersonal cultural empathy, with some influence on interpersonal cultural empathy. Students were highly satisfied with this learning experience	♦ Empathy skill ♦ Major-related knowledge	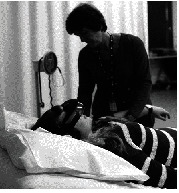
Silverman et al., USA. [46].	To develop and test a novel impairment simulation activity to teach beginning rehabilitation students how people adapt to physical impairments	Experiential learning and the control group: (video-viewing)	Postparaplegia and hemiplegia rehabilitation training	Occupational therapy and physiotherapy students	Randomized clinical trial	*n* = 32 OT: 4, DPT = 18 Sex = (*F* = 78%, *M* = 22%) Age = 20-43 y Mean: 26.94 y	Experimental activity more positively (*M* = 4.63, SD = 0.50) than the control activity (*M* = 4.00, SD = 0.53; *t* = 3.36, *p* = 0.0022).	Impairment simulation can be an effective way to teach rehabilitation students about the adaptations that people make to physical impairments. Positive impairment simulations should allow students to experience success in completing activities of daily living with impairments. Impairment simulation is complementary to other pedagogical methods, such as simulated clinical encounters using standardized patients	♦ Major-related knowledge ♦ Skills	Without picture
Liaw et al., USA. [37].	To evaluate healthcare students' perspectives on the transferability of the IPE virtual reality simulation learning to clinical practice	Simulation scenarios	Interprofessional skills	Occupational therapy and physiotherapy students	Qualitative design	Medical (*n* = 7) Nurse (*n* = 5) PT (*n* = 2) MSW (*n* = 2) Sex = (*F* = 56%, *M* = 44%) Age = 23.7 y (SD = 1.81)	Gaining insights into mutual roles and seeing the patient as a whole gap in real-world application	Early exposure to team care through IPE virtual reality simulation can foster an understanding of the interdependent roles of healthcare professionals toward patient-centered care. For greater clinical impact, a further recommendation is to supplement with workplace-based team training to contextualize learning with practice settings	♦ Communication interprofessional	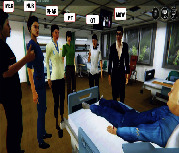
Liaw et al., USA. [47].	To describe the development of a 3D-VE and to evaluate healthcare students' experiences of their collaborative learning in the environment	Three dimensional virtual environments (3D-VE)	Interprofessional skills	Occupational therapy and physiotherapy students	A mixed methods study	MED (*n* = 6) NUR (*n* = 6) PHAR (*n* = 4) PT (*n* = 6) OT (*n* = 6) MSW (*n* = 1) Sex = (*F* = 22, *M* = 14) Age = 21-23 y	The students demonstrated significant improvements in their attitudes towards healthcare teams (*p* < 0.05) and interprofessional collaboration (*p* < 0.001) after the collaborative learning	Given its flexibility, practicality, and scalability, this 3D-VE serves as a promising tool for collaborative learning across different healthcare courses and institutions in preparing for future collaborative-ready workforces	♦ Communication interprofessional	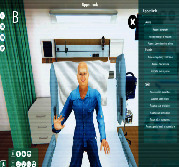
Silén et al., Sweden. [48].	To support learning efficacy by developing and using 3D datasets in regular healthcare curricula and enhancing the knowledge about possible educational value of 3D visualizations in learning anatomy and physiology	3D visualization: QuickTime VR	Healthcare students (anatomy and physiology)	Healthcare students	Clinical trial	*n* = 62 (MDs, PTS) Age = 21-23 y	Visualizations with varying degrees of interactivity, produced by modern medical imaging equipment, are a promising resource in student-centered medical education	3D visualizations based on authentic, viable material point out a new dimension of learning material in anatomy, physiology, and probably also pathophysiology. It was successful to implement 3D images in already existing themes in the educational programs. The results show that deeper knowledge is required about students' interpretation of images/films in relation to learning outcomes. There is also a need for preparations and facilitation principles connected to the use of 3D visualizations	♦ Observational Skills ♦ Theoretical learning	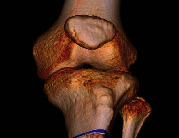
Ulrich et al., Denmark. [49].	To explore the learning effectiveness of 360° video when used as e-learning for healthcare students and whether the technology is a good IT investment for education institutions	Virtual reality with 360° video and head-mounted display: VR-HMD	Practical skills training	Physiotherapy students	Before and after trial	Frst group (*n* = 28) (VR-HMD). Second group (*n* = 26) used regular video shown on a laptop Third group (*n* = 27) received traditional teaching absent of technology use	The paired-sample *T*-test determined whether there was a significant difference in the academic results between the ex-posttest (*M* = 6728 test score, SD = 1173) opposed to the pretest (*M* = 2469 test score, SD = 1534). The *T*-test demonstrated that the students obtained a mean increase of 4.259 in the test scores, with 95% CI (3.832, 4686), *t* (80) =19.875, *p* < 0.000	The results show 360° video to be equally effective compared to regular video but less effective than traditional teaching. Moreover, 360° video performed better than regular video in the students' emotions about the technology. We find that 360° video could be a viable alternative to video in e-learning for healthcare students	♦ Learning practice change	Without picture
Kelly et al., Australia. [50]	Augmented studio is an augmented reality system that helps facilitate this by projecting anatomical structures over moving bodies and allowing annotation of these structures	Augmented reality technology (augmented studio): projection mapping to display anatomical information, such as bones and muscles, on the human body in real time whilst the body is moving	Pedagogical practices in physiotherapy in teaching of manual skills	Physiotherapy students	Case-control study	PTS *n* = 9 Sex = (*M* = 3, *F* = 6) Age = 21-29 y (mean 24.7, SD 2.27) First *y* = 2, second *y* = 3, final Three teachers also participated in the evaluation	It can be seen that augmented studio received a positive response on the majority of factors (mean > 4.0). Overall: assist learning ($\overset{¯}{x}$: 4.3, SD: 0.48), satisfaction ($\overset{¯}{x}$: 4.1, SD: 0.57), self-rated success ($\overset{¯}{x}$: 4.1, SD: 0.57). Experience: better than traditional class ($\overset{¯}{x}$: 3.9, SD: 0.57), enjoyment ($\overset{¯}{x}$: 4.6, SD: 0.52), improves communication ($\overset{¯}{x}$: 4.3, SD: 0.67).Use intention: (improves understanding of anatomical structures ($\overset{¯}{x}$: 4.6, SD: 0.52), kinesiology ($\overset{¯}{x}$: 4.1, SD: 0.74), anatomy movement ($\overset{¯}{x}$: 3.7, SD: 0.95)	Outcomes from a pilot usability study showed that augmented studio promotes the creation of an engaging teaching and learning experience and the facilitation of communication between teachers and students	♦ Communication interprofessional ♦ Learning	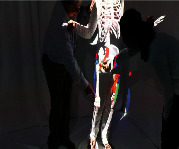
Miller-Cribbs et al., USA. [51].	The professional ACE-informed training for health! (PATH!) educational program and simulation experience using standardized patients (SP) was developed to help healthcare professionals address ACEs with adults	Video-based training program: CAE learning space clinical simulation platform	To help healthcare professionals address ACEs with adults	Occupational therapy and physiotherapy students	Preliminary evaluation	*n* = 53 learners participated OT master's, PT doctoral students (*n* = 15), family medicine or internal medicine residents (*n* = 38) (years 1 through 4	The most frequently coded learner behaviors or actions were asking open-ended general questions (*F* = 279), missing a cue to respond empathetically (*f* = 169), asking a close-ended question about ACEs (*f* = 112), taking a cue to engage in collaborative treatment planning (*f* = 106), taking a cue to explain ACEs to the patient (*f* = 77), responding to/or addressing stigma (*f* = 74). SP actions most frequently coded were nonverbal responses to learner (*f* = 511), verbal responses to learner (*f* = 220), questions about ACEs (*f* = 88), disclosing ACE history (*f* = 86), and acknowledgement of ACEs (*f* = 24)	That medical residents and OT and PT students demonstrated skills during SP encounters congruent with TI training on addressing ACEs with adults, particularly in explaining ACEs, demonstrating empathy, collaborative treatment planning, and stigma reduction. PC residents showed both positive and negative changes in PATH!-specific skills from year 1 to 4 of the training program. This study supports the PATH! model and simulation-based training in preparing clinicians to address ACEs with adults and provides insight into further curriculum improvement	♦ Interpersonal skills Attitudes ♦ Observational skills	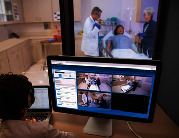
Hough et al., Australia. [11].	To investigate the effect of SBE on student self-efficacy in the physiotherapy assessment and management of paediatric clients, and to determine student satisfaction with SBE as a learning strategy	Simulation-based education (SBE)	Pediatric clinical training	Physiotherapists	Observational study	PTs *n* = 92 (2EXCUD) pre- and post-SBE questionnaires for SBE scenario 1 (*n* = 86/90, 96%) Both pre- and post-SBE questionnaires for scenario 2 (*n* = 82/90, 91%) Both pre- and post-SBE questionnaires for scenario3 (*n* = 77/90, 86%) Students completed the learning reactionaries. (*n* = 77/90, 86%)	This study, self-efficacy survey response rate varied from 77-96% for each session. Significant increases in mean student self-efficacy were recorded for all questions (*p* < 0.001). A total of 139 (85%) responded to the learning reactionaries with 78.6% indicating they were very satisfied with SBE as a learning strategy	The results of the self-efficacy questionnaire were clearly positive, with significant improvements to 13 student self-efficacy post-SBE recorded for every question, indicating that SBE has a positive effect on student self-efficacy in the physiotherapy assessment and management of pediatrics clients. Students also reported that they found SBE to be a valuable learning experience	♦ Physiotherapy student self-efficacy	Without picture
Hartstein et al., USA [52].	To compare the effects of virtual reality (VR) patient simulation with those of a traditional standardized patient simulation on the CDM of student physical therapists (SPTs)	Virtual reality (VR) patient simulation	Clinical decision-making (CDM) skills	Physiotherapy students	A randomized controlled trial	PTs *n* = 59 Sex = (20 M, 39 F) Age = (mean 24.02, SD 2.73)y First group: virtual reality instructional design *n* = 30 Second group; standard patient design *n* = 29	Statistically significant within group differences in CDM were noted after both VR and standardized patient instruction, but no between-group differences were found. The effect sizes were considered large with either learning experience; the observed experimental effect was greater after a VR experience. No between-group differences were found between metacognitive awareness, diagnostic accuracy, or psychomotor skill assessment. Diagnostic efficiency was statistically significantly greater in the standardized patient condition, while engagement was significantly greater in the VR condition	The instructional benefits of immersive VR learning match the needs of first-year students and provide opportunities to explore the outcomes of various clinical decisions, confront assumptions, and receive feedback in a repeatable and low-stakes learning environment. These results suggest that both VR and standardized patient learning experiences may be beneficial to the development of CDM skills among SPTs.	♦ Clinical decision-making skills	Without picture

Applied abbreviations: F: female; M: male; PTs: physiotherapy student; OTs: occupational therapy student; DPT: Doctor of Physiotherapy; RN: students in nursing; PT: physiotherapy; RT: respiratory therapy; SD: standard deviation; MSW: medical social work; PHAR: pharmacy; NUR: nursing; VR-HMD: virtual reality head-mounted display; MIKAT: Motivational Interviewing Knowledge and Attitudes Test; MITI: Motivational Interviewing Treatment Integrity scale; TP1: pretraining immediately; TP2: posttraining (immediately following completion of student's subsequent placement posttraining); IREX: The Gesture Tek Interactive Rehabilitation Exercise System; ST: systems thinking; STS: systems thinking scale, FNER: Friday Night at the Emergency Room.

**Table 2:** Risk of bias summary; three qualitative papers assessed by the JBI Checklist.

+	+	+	Is there congruity between the stated philosophical perspective and the research methodology?
+	+	+	Is there congruity between the research methodology and the research question or objectives?
+	+	+	Is there congruity between the research methodology and the methods used to collect data?
+	+	+	Is there congruity between the research methodology and the representation and analysis of data?
+	+	+	Is there congruity between the research methodology and the interpretation of results?
+	+	+	Is there a statement locating the researcher culturally or theoretically?
—	—	—	Is the influence of the researcher on the research, and vice-versa, addressed?
—	—	—	Are participants, and their voices, adequately represented?
+	+	+	Is the research ethical according to current criteria or, for recent studies, and is there evidence of ethical approval by an appropriate body?
+	+	+	Do the conclusions drawn in the research report flow from the analysis, or interpretation, of the data?
Ref. [42]	Ref. [20]	Ref. [37]	

Scores: (+): yes (low risk of bias), (?): unclear, and (-) no (high risk of bias).

**Table 3:** Distribution of studies by study populations and scope of education.

Row labels	Frequency	% of total
Physiotherapy students	7	43.7%
Cardiopulmonary hearing skills training	1	6.2%
Clinical decision-making skills	1	6.2%
Cultural empathy training	1	6.2%
Nervous physiotherapy training	1	6.2%
Pediatric clinical training	1	6.2%
Practical skills training	2	12.5%
Physiotherapy and occupational therapy students	2	12.5%
Interprofessional skills	2	12.5%
Masters of occupational therapy students and doctor of physical therapy students	1	6.2%
Postparaplegia and hemiplegia rehabilitation training	1	6.2%
Healthcare students with physiotherapy students	1	6.2%
Healthcare students (anatomy and physiology)	1	6.2%
Students from six healthcare courses in medicine, nursing, pharmacy, physiotherapy, occupational therapy, and social work formed	1	6.2%
Interprofessional skills	1	6.2%
Medicine, nursing, pharmacy, physiotherapy, occupational therapy students	1	6.2%
Interprofessional skills	1	6.2%
Students of the Doctor of Physiotherapy	1	6.2%
Pedagogical practices in physiotherapy in teaching of manual skills	1	6.2%
Nursing, physiotherapy, medicine, and occupational therapy students	1	6.2%
Interprofessional skills	1	6.2%
Occupational therapy, physiotherapy, and medicine students	1	6.2%
To help healthcare professionals address ACEs with adults	1	6.2%
Grand total	16	100%

**Table 4:** Effects of computerized simulation education on outcomes.

Outcome category	Outcomes subcategory	Effect	
		Positive	
		SS	EWA
Professional skills, behaviors, and knowledge's effectiveness	Health behavior	1 (6.2%)	—
	Communication interprofessional	3 (17.7%)	3 (17.7%)
	Practical skills	3 (17.7%)	1 (6.2%)
	Personal behavior and attitudes	1 (6.2%)	1 (6.2%)
	Physical awareness	1 (6.2%)	—
	Empathy skill	2 (11.5%)	—
	Theoretical learning	1 (6.2%)	1 (6.2%)
	Major-related knowledge	2 (11.5%)	1 (6.2%)
	Interpersonal skills	2 (11.5%)	—
	Observational skills	2 (11.5%)	2 (11.5%)
	Clinical decision-making skills	1 (6.2%)	—

Physiotherapist-reported effectiveness	Learning practice change	2 (11.5%)	—
	Confidence	1 (6.2%)	—
	Physiotherapy student self-efficacy	1 (6.2%)	—
	Reducing the dependency on educators	1 (6.2%)	1 (6.2%)

SS: statistically significant; EWA: effective without arguments.

**(a):** 

#	Reported limitations of conducting research	Studies
1	Small sample size in groups	[8, 31, 42, 44, 46, 48, 49, 51, 52]
2	Lack of interaction	[20, 37, 42, 45]
3	Short follow-up duration	[8, 42, 46]
4	The evaluation on the transferability of the IPE education is subjective	[37, 44, 47]
5	Nonstandardized survey used	[36, 43, 44]
6	Selection not covered all students	[46]
7	There is no comparison to standard education methods	[11]
8	Only undergraduate university students were included in this study, so results should not be extrapolated and applied to other populations such as junior, employed physiotherapists	[11]
9	Lack of blindness of participants	[8, 52]
11	Asynchronous control and experimental blocks	[8]
12	Assessment solutions are not approved by home care therapists	[36]
13	Lack of experience	[20]
14	Failure to record the results of psychometric properties in PT students	[45]

**(b):** 

#	Barriers to the use of technologies	Studies
1	Cost of the technology in terms of time, money, space, and implementation	[11, 50–52]
2	Need sufficient space	[8, 36, 49]
3	System's inflexibility	[8, 42]
4	Simple system design for quick access and easy use	[8]
5	Lack of long-lasting effects	[11]
6	Need to simulate software management platform	[51]
7	Difficult to develop and sustain virtual programs	[50]
8	Need to different learning situations	[48]
9	Need time to become familiar with the technology	[43]
10	Bandwidth limitation	[47]
11	Emphasize specific competencies in using the equipment	[46]
12	Problems with handling the network	[47]
13	Uncertain accessibility to online training	[8]
14	The system currently needs a technician	[50]
15	VR technology may threaten the external validity	[52]

## Data Availability

All data generated or analyzed during this study are included in this published article.

## Supplementary Materials

Supplementary Materials Supplementary Materials Table S1: the filled PRISMA 2020 Checklist is provided. Table S2: the search strategies for PubMed and Scopus databases are given.
